# Retraction: Capturing carbon dioxide as a polymer from natural gas

**DOI:** 10.1038/ncomms13456

**Published:** 2016-11-01

**Authors:** Chih-Chau Hwang, Josiah J. Tour, Carter Kittrell, Laura Espinal, Lawrence B. Alemany, James M. Tour

Nature Communications 5: Article number: 3961; DOI: 10.1038/ncomms4961 (2014); Published: 06 03 2014; Updated: 11 01 2016

In this Article, we reported the synthesis of nucleophilic sulfur- and nitrogen-containing porous carbons and their carbon dioxide uptake performance. Specifically, we described a mechanism where the carbon dioxide polymerized in the channels of the porous support. Since the publication of the Article we have been unable to reproduce the infrared, Raman and solid-state ^13^C-NMR spectra that supported this mechanism; these data had been generated in the laboratory of J.M.T. During our efforts to reproduce the infrared spectra, the band centred at 1,735 cm^−1^ was not as pronounced as in the published data. The Raman band at 798 cm^−1^ was observed on the occasion but disappeared with further focusing of the instrument; and the 166.5 p.p.m. peak in the solid-state ^13^C-NMR could not be acquired again. While the gas adsorption data are without question and reproduced across laboratories, we are no longer confident in the spectroscopic data supporting the proposed carbon dioxide polymerization mechanism and we, the authors, therefore wish to retract this Article.

